# Pregnancy loss: Consequences for mental health

**DOI:** 10.3389/fgwh.2022.1032212

**Published:** 2023-01-23

**Authors:** Diana Cuenca

**Affiliations:** Hospital of Torrejón, Torrejón de Ardoz, Spain

**Keywords:** mental health, abortion, abortion—spontaneous, stillbirth, miscarriage, fetal reduction

## Abstract

Pregnancy loss, in all its forms (miscarriage, abortion, and fetal death), is one of the most common adverse pregnancy outcomes, but the psychological impact of such loss is often underestimated. The individual response to this outcome may vary between women—and could be influenced by age, race, culture, or religious beliefs—but most experience anxiety, stress, and symptoms of depression. Because pregnancy loss is not uncommon, health providers are used to dealing with this diagnosis, however the correct management of the process of diagnosis, information-gathering, and treatment can greatly ameliorate the adverse mental consequences for these women. The aim of this review is to examine the different types of pregnancy loss, and consider how each can influence the mental health of the women affected and their partners—in both the short- and long-term; to review the risk factors with the aim of identifying the women who may be at risk of consequential mental health problems; and to provide some advice for health providers to help these women better cope with pregnancy loss. Finally, we provide some points for health providers to follow in order to aid the management of a pregnancy loss, particularly for spontaneous, induced, or recurrent miscarriage, or stillbirth.

## Introduction

A pregnancy loss, in all its forms, whether it miscarriage, abortion, or fetal loss, is today one of the most common adverse pregnancy outcomes today. In many cases pregnancy loss is associated with short- and long-term psychological effects, which are often underestimated by health professionals (1). The response to such loss has changed over time, and can be influenced by factors that include religious and cultural issues, age, and stage of pregnancy. However, there will surely always be some psychological impact, whether mild or severe.

Pregnancy loss can occur spontaneously or voluntarily. Induced abortion is one of the most common surgical interventions worldwide, and its psychological repercussions are not negligible. These are influenced by many factors that could not be excluded from this review. The frequency of miscarriage worldwide also makes its psychological impact significant. The aim of this review is: first, to analyze the psychological morbidities associated with the discrete types of gestational loss; second, to consider the associated risk factors and protective factors with the aim of identifying those who might be at risk of experiencing negative mental health outcomes; and third, to provide some advice and recommendations for the healthcare providers involved in prenatal care to aid the detection of those at risk in order to prevent or reduce adverse psychological outcomes.

## Classification of pregnancy loss

There is no consensus in the classification of pregnancy loss, with definitions differing around the world. In the absence of such consensus, we propose a classification of gestational loss, which is shown in Table 1. We employ this classification in the following exposition.

**Table 1:** Classification of pregnancy loss.

Spontaneous abortion or miscarriage	Early pregnancy loss, miscarriage, or spontaneous abortion	Before 10 weeks of pregnancy (2)
	Late abortion, late miscarriage, or fetal miscarriage	Between 10 and 22 weeks of pregnancy (2)
	Recurrent miscarriage	Two or more (3) or three or more (4) miscarriages not required to be consecutive; or two or more consecutive miscarriages (5)
	Fetal loss or stillbirth	After >22 weeks (6)
Voluntary termination of pregnancy	Abortion or induced abortion	Voluntarily termination of pregnancy, including termination due to fetal malformations (7)
	Fetal reduction	Specific type of induced abortion involving a multiple gestation, where one or more of the fetuses are chosen to be voluntarily terminated (8)

We can classify spontaneous abortion, or miscarriage, by early and late abortion. We use the term “early pregnancy loss” for those occurring before 10 weeks of gestation (2). Early pregnancy loss is defined as a nonviable, intrauterine pregnancy with either an empty gestational sac or a gestational sac containing an embryo without fetal heart activity within the first 10 + 6 weeks of gestation (2). We use the term “late abortion,” “late miscarriage,” or “fetal miscarriage” when for those occurring between 10 and 22 weeks of gestation (crown–rump length > 33 mm) (2).

The term “recurrent miscarriage” is harder to define, for example, we can use this term when a spontaneous loss occurs in: at least two gestations, not required to be consecutive (3); three or more gestations, not required to be consecutive (4); or two or more consecutive gestations (5).

We use the term “fetal loss,” or “stillbirth,” when the pregnancy loss occurs after 22 weeks of gestation (6).

We use the term “induced abortion” when a controlled procedure is performed in order to terminate a pregnancy. Reasons to terminate a pregnancy include an unplanned gestation, fetal malformation, and maternal health complication (7).

“Fetal reduction” is a specific type of induced abortion involving a multiple gestation, where one or more of the fetuses are voluntarily selected for termination (8).

## Spontaneous pregnancy loss: mental health consequences

### Early and late pregnancy loss

Studies indicate that approximately 20% of all pregnancies end in spontaneous abortion within the first 22 weeks (9), making early pregnancy loss the most common obstetric complication. However, this high incidence rate can lead to health providers (and society in general) underestimating the impact on the mental health of those affected. The high incidence rate also means that early miscarriage is often overlooked in research (9).

It is easy to assume that the later the loss of a pregnancy, the more repercussions on the woman's physical and mental health (10). A number of variables have been cited as influencing the way patients and their partners cope with pregnancy loss, acting as protective or risk factors for adverse mental health outcomes (11).

Some studies have reported the deleterious effects of spontaneous abortion on women's mental health: 55% of women presented symptoms of depression, up to 27% perinatal grief; and more than 18% reported moderate anxiety (11). Some of the risk factors that have been cited for presenting depression, anxiety and perinatal grief are:

– low socioeconomic status (11, 12),
– childless women (11, 12),
– low standard of education(13), and
– fewer than 6 months since miscarriage (13).

Research suggest that race can also affect mental consequences (14). Black women who are undergoing early pregnancy loss treatment, are about twice as likely to experience risk for major depression compared with non-Black women, a reason to establish black race as a risk factor for adverse outcome.

Some of the protective factors that have been cited in the prevention of depression, anxiety and perinatal grief are

– high standard of education (11, 15),
– non-immigrant women (14),
– good conjugal relations (11, 16), and
– satisfaction with healthcare received (11, 16).

### Recurrent miscarriage

Recurrent miscarriage is broadly defined as the repeated, unintentional loss of pregnancies. Depending on the definition used the prevalence is different. If two or more pregnancy losses are used as the definition of recurrent miscarriage, the prevalence is to 2%–6% of the general population, but this decreases to 0.7% if we use the definition of three or more pregnancy losses (3–5, 17). This figure includes only hospital-treated miscarriages, so the true prevalence in the general population may be higher.

Repeated pregnancy loss results in feelings of discouragement and emotional pain. Many psychological disorders have been described in those who experience recurrent pregnancy loss, such as increased risk of anxiety, depression, grief, guilt, and anger (18). Symptoms of moderate or severe depression are present in about 10% of affected couples, and high stress levels are reported in more than 40% of patients with recurrent pregnancy loss (19).

Compared to women with only one prior miscarriage, couples with recurrent pregnancy loss present severe symptoms of both depression and stress, for a longer time, and experience a greater negative impact on their mental health during subsequent pregnancies (20).

Recurrent miscarriage affects not only the woman who miscarries but also her male partner. If we compare the effects of recurrent miscarriage, 72.7% of the affected women and 66.3% of their male partners revealed a risk of anxiety; and 51% of women and 19% of their male partners presented a risk of depression (21–23). In general, the risks of experiencing depression and anxiety are higher in women.

Some risk factors for the psychological effects on couples experiencing recurrent miscarriage are: limited social support and the number of previous pregnancy losses (21).

Two of the main protective factors for adverse outcomes are: a previous child with the current partner, and the couple’s perceived satisfaction with their relationship (21).

A flexible system must be put into place to help the affected people cope with recurrent losses, and to ease any frustrations or doubts about their reproductive abilities (9).

General recommendations include: allowing greater recovery time (optimal recovery time varies on a case-by-case basis); accepting each other’s feelings; receiving support from others; and asking for advice from professional health providers if necessary (24).

Recurrent miscarriage can adversely affect mental health during future pregnancies. In addition, those who experience recurrent miscarriage are generally dissatisfied with the medical care they receive and want a more individualized approach to care (24, 25). They require sensitivity, empathy, acknowledgment of their losses, and access to accurate and reliable information about the etiology of their loss. The majority also perceive lack of follow-up from medical staff (25, 26).

The concept of “tender loving care” reoccurs in some literature, advocating for increased emotional support and increased monitoring for those who have previously experienced recurrent miscarriage. This recommendation is based on results which document successful pregnancy outcomes in 86% of women who received this level of supportive care and close monitoring, compared with only 33% successful pregnancy outcomes for women who did not receive this level of attention (25, 27).

### Stillbirth

Approximately 2% of all pregnancies end in stillbirth. This is defined as an intrauterine fetal death occurring after 22 weeks (6). Stillbirth is an extremely significant adverse pregnancy outcome affecting both short- and long-term psychological wellbeing (28). It can be argued that the effects of stillbirth cannot be compared with other, earlier gestational losses because the prospective parent is losing a person who is already part of their life, with whom they have planned to spend time, and with whom they have imagined a future together (29). In some cultures, the mother is blamed for the stillbirth, leading to social stigmatization. It is very common for those who suffer a stillbirth to experience feelings of guilt and shame, compounded by an inability to express their grief over the loss, which in turn exacerbates rates of depression. Many who suffer stillbirth socially isolate themselves, thereby aggravating short- and long-term depressive symptoms (30, 31).

In approximately 50% of stillbirths the etiology is undeterminable (32), further contributing to a sense of there being a lack of answers and compounding feelings of guilt and shame. For many who have lost a child, the care they receive after the event will have consequences for their self-esteem and even their self-identity, it is therefore important to comprehend the different phases in the process of perinatal grief, and how health providers might help people coping with such loss (33).

Different stages of perinatal grief may be defined as: shock at the moment of diagnosis; disorientation; the phase of maximum sadness; emptiness and helplessness; reorganization; and finally acceptance (25).For the patient the first moment after the diagnosis is the shock phase: the patient has to accept the clinical news. The main characteristics of this phase are denying the news; being non-responsive to stimulus; and, after that, having feelings of anger. Despite this, the patient has to deal with giving birth to a baby who has died and they will naturally feel fearful of the process. In the very first moments they will feel pain, but after that their anger will focus on what they can blame (30).

It is in this first phase that psychosocial support by medical providers occurs. The way medical providers act and manage this situation has the potential to significantly improve a patient's outcome after stillbirth and can help minimize the negative risks of traumatic grief (33).

In the long term those who have had a stillbirth are likely to experience adverse mental health. Anxiety and depression during the first months are common in up to 50% of couples, with the effects being present for up to 3 years after the event (27), which is much longer than other gestational losses. The phase of reorganization and acceptance, involving a return to normal life, usually begins 18 months after the loss, although this varies according to personal circumstances.

Despite the acceptance phase having passed, these couples continue to exhibit psychological symptoms in other aspects of life. A case–control study carried out at Oslo University Hospital, comparing mental health and quality of life of women after stillbirth with women after healthy deliveries, concluded that the former more frequently reported pain, were more physically and mentally exhausted from work, and felt that their health was worse (33, 34).

The study also found that those cases report a slightly lower quality of life, and that this fact does not change with time elapsed since the loss (less or more than 10 years) (34). In addition, poorer marital relationships, disharmony, and sexual problems are also more common in these couples.

The risk of experiencing depression, anxiety, and post-traumatic stress disorder in a subsequent pregnancy is higher after a fetal death, and this risk is greater the shorter the time since the loss, although these symptoms often decrease postnatally (35, 36). Screening for adverse psychological issues is appropriate at the first and at any follow-up visits in order that appropriate medical support can be provided.

Data on the management of pregnancies after an unexplained stillbirth are scarce. Women should be encouraged to minimize the risks of stillbirth that are attributable to modifiable risk factors such as optimizing glycemic control, avoiding smoking, following a healthy diet (37). However, a 2018 Cochrane review found insufficient evidence to issue a clinical practice guide including effective interventions to improve care for women with a history of stillbirth (38).

## Voluntary termination of pregnancy: mental health consequences

### Induced abortion

Elective abortion is one of the most common medical interventions worldwide, with approximately 73 million induced abortions performed each year (39).

An unintentional pregnancy brings emotional stress: with the woman having to decide how to deal with the pregnancy and what to do in a very short period of time, and then experiencing an induced abortion. There are consequences for the mental health of the women that undergo voluntary termination (40). Due to liberal abortion laws, 15% of the world’s elective abortions are performed in China. A study performed in China, with the aim of determining the prevalence of stress and depression after an induced abortion, concluded that while some women cope well with the process, others suffer a range of mental health issues. The overall prevalence of high perceived stress was 25.3% and the rate of depression 22.5% (40)—a lower rate than in the case of miscarriage (11).

Numerous risk factors can help identify which women might be at risk of negative psychological outcomes after an induced abortion. Improved screening for these risk factors should help identify women who might benefit from additional pre- or post-abortion counseling (41). We classify these risk factors as being associated with: social support, previous mental health issues, and clinical factors (42):

– **Associated with social support:** perceived pressure from others to terminate a pregnancy; perceived opposition to the abortion from partner, family or friends; feelings of stigma or the need for secrecy have been described as risk factors for developing depression and trauma (43, 44).
– **Associated with previous mental health issues:** a prior history of mental health problems can have a negative impact for women that decide to terminate a pregnancy. Personality factors, such as low self-esteem and low perceived control over their own life, can also increase the possibility of developing depression and stress disorder. In terms of behavior, denial of the process, avoidance, and doubts about the decision they have taken all have a negative impact on the future mental health of these women (45).
– **Associated with clinical factors**: a prior history of abortion or an abortion after the first trimester have been identified as risk factors for adverse psychological outcomes.

Having identified these risk factors, there have been numerous recommendations for abortion providers to screen patients with the aim of providing additional counseling for those who need it—both before an abortion, including decision-making counseling, and afterward in the form of a follow-up (42). Doing so could be very effective in reducing long-term adverse results.

However, a systematic review that explored the effect of psychological and supportive interventions in reducing stress levels in women with a history of induced abortion concluded that there is no evidence of psychological support being effective in the prevention of adverse outcomes (45).

### Fetal reduction

Fetal reduction is an abortion performed when elective fetal termination is indicated for triplets, quadruplets, and other higher order multiple pregnancies; or when a fetal defect is present in one of the fetuses in a multiple pregnancy (46). There is balance between the elective abortion and the birth of the surviving fetus, making this a nuanced procedure. Although it is suggested that patients involved in fetal reduction may experience negative mental health consequences, in fact the procedure is considered necessary in order to improve the health of the surviving fetus, and so the negative psychological effects are different and less severe compared with other types of pregnancy loss (46).

Studies have found that feelings of sadness and depression are very common on the day of the termination, affecting up to 69% of couples (47). More than half of the couples were anxious about the pain and the procedure itself; and 57% of subjects reported guilt on the day of the reduction (8). Despite these high percentages, in the medium and long term these patients experience a much lower rate of negative mental outcomes than those receiving an induced abortion in a single-fetus pregnancy (8, 49). In general, fetal reduction is considered to be well-tolerated.

One psychological effect, unique in this kind of abortion, is that the majority of patients tested (88.2%) describe fantasies about the terminated fetuses after the birth of the surviving baby, and even months later (48).

Finally, when the procedure of fetal reduction results in the loss of all fetuses in a multiple pregnancy the rate of depression is one of the highest described, in up to 75% of the couples (49). This situation is associated with a feeling of guilt for what has happened and of taken the wrong decision which increases the symptoms of pathological perinatal grief.

## Consequences for future postpartum mental health

A prospective cohort study including more than 1,900,000 women compared the need for postpartum psychiatric treatment within 6 months of the delivery of their first live birth in women with a prior history of pregnancy loss (spontaneous or induced), with women with no history of pregnancy loss (50). Women with one or more pregnancy losses prior to their first live births were approximately 35% more likely to require psychiatric treatment than women without a history of pregnancy loss. As well as pregnancy loss a history of prior mental health treatment, independently but especially in combination with a prior pregnancy loss, is a risk factor in the need for postpartum psychiatric treatment (50, 51). Because psychiatric treatment is reserved for women with moderate to severe symptoms, this study enables us to focus on women at high risk of major depression and with high levels of anxiety.

## Consequences for mental health in future pregnancies

As we have already established, miscarriages are associated with increased levels of distress, anxiety, and depression in the population concerned (11, 12). Psychological symptoms, such as stress and depression, have been linked to adverse pregnancy outcomes, including preterm labor and low birth weight (52). Therefore, identifying women at risk, and designing appropriate interventions in order to prevent such symptoms during pregnancy, can reduce adverse effects not only in the months after the miscarriage but also during the course of any future pregnancies. The effect of psychological management in these cases and future consequences has not been studied in detail, so, as well as in cases of spontaneous loss, more studies are needed in this regard (29).

## Management of pregnancy loss

When a pregnancy loss occurs, either spontaneous or induced, the correct intervention and support in the first phase of perinatal grief is associated with a lower depression rate and a better quality of life in the future (53). It is therefore important that health providers that assist these patients know the correct way to manage these situations.

General recommendations for managing pregnancy loss are applicable for spontaneous or induced abortion, recurrent miscarriage, and stillbirth (54), although stillbirth has some additional, specific recommendations.

Based on the needs expressed by participants in a study, we have created a list of recommendations for health providers and steps to follow (53):

– Health professionals, physicians and midwives must be familiar with the tools for first-moment management after the diagnosis of a pregnancy loss in order to understand the situation, as well as knowing how to help and support these patients. The care provided should be individualized, preferably delivered by the same staff that were present at the diagnosis (53, 55).
– Health professionals must take time to empathize, give emotional support, and answer questions posed by the couple in order to comfort them. Communication must be clear prior to making an informed decision about any proposed treatment; and give the couple the opportunity to openly talk about their feelings and doubts (55).
– Terms such as “miscarriage” may be hurtful or considered insensitive, so should be avoided. Instead use terms such as “baby,” “your child,” or the name of the lost baby (56, 57).
– During the process the partner should not be excluded from receiving compassionate care and adequate information (58).
– A private area should be provided for the couple and their families, away from pregnant women (53, 59).
– Specifically in the case of stillbirth, much research evidence suggests that contact with the baby after the birth was associated with a lower risk of depressive and anxious symptoms, both during the perinatal period and in a subsequent pregnancy (60). This should be actively recommended by health providers.

There are some actions and interventions carried out by health providers that must be avoided (60):

– Some depressant drugs that can be used to relax a couple in shock in the first-moment phase, and can enable a feeling of negation of the situation. However in the future they can have an adverse effect for the process of acceptance (53).
– Health providers should not avoid talking about the gestational loss. Although it is a painful and uncomfortable process for all involved, avoidance behaviors can make the couple feel that the loss is not significant (53).
– Avoiding contact between parents and baby, or hiding the baby's items, only exacerbate the fantasies the couple create about the lost baby. Delaying contact with the baby can extend the state of denial (61).
– Avoid expressions like: “You'll get pregnant again,” “you will have more children,” or “at least it happened before the baby was born and not after,” “you are still young”. These will only compound the affected couples’ sense of being misunderstood.

## Conclusions

Pregnancy loss is the most common adverse outcome for women expecting a baby. Although different types of gestational loss may have common features, each can affect the future mental health of couples in different ways. It is important for clinicians to be familiar with factors in the development of adverse mental outcomes. In doing so, they can act appropriately at the point of diagnosis and treatment. In addition, they can aim to identify, early on, those women who might be at risk of developing an adverse mental outcome, in order to implement proper monitoring.

## Author contributions

DC is the only author and has carried out the whole review process.
