# Corrigendum: Pregnancy loss: consequences for mental health

**DOI:** 10.3389/fgwh.2023.1266931

**Published:** 2023-08-24

**Authors:** Diana Cuenca

**Affiliations:** Hospital of Torrejón, Torrejón de Ardoz, Spain

**Keywords:** mental health, abortion, abortion—spontaneous, stillbirth, miscarriage, fetal reduction

A Corrigendum on Pregnancy loss: consequences for mental health By Cuenca D (2023). Front. Glob. Womens Health 3:1032212. doi: 10.3389/fgwh.2022.1032212

**Text Correction**

In the published article, there was an error. What the authors wanted to focus on in the text was not expressed correctly.

A correction has been made to **Consequences for mental health in future pregnancies,** paragraph 1. The sentence previously stated:

“Therefore, identifying women at risk, and designing appropriate interventions in order to prevent such symptoms during pregnancy, can reduce adverse effects not only in the months after the miscarriage but also during the course of any future pregnancies (29).”

The corrected sentence appears below:

“Therefore, identifying women at risk, and designing appropriate interventions in order to prevent such symptoms during pregnancy, can reduce adverse effects not only in the months after the miscarriage but also during the course of any future pregnancies. The effect of psychological management in these cases and future consequences has not been studied in detail, so, as well as in cases of spontaneous loss, more studies are needed in this regard (29).”

The authors apologize for this error and state that this does not change the scientific conclusions of the article in any way. The original article has been updated.

